# Olfactory Ensheathing Cell Transplantation in Experimental Spinal Cord Injury: Effect size and Reporting Bias of 62 Experimental Treatments: A Systematic Review and Meta-Analysis

**DOI:** 10.1371/journal.pbio.1002468

**Published:** 2016-05-31

**Authors:** Ralf Watzlawick, Julian Rind, Emily S. Sena, Benedikt Brommer, Tian Zhang, Marcel A. Kopp, Ulrich Dirnagl, Malcolm R. Macleod, David W. Howells, Jan M. Schwab

**Affiliations:** 1 Department of Neurology and Experimental Neurology, Charité Campus Mitte, Clinical and Experimental Spinal Cord Injury Research Laboratory (Neuroparaplegiology), Charité–Universitätsmedizin Berlin, Berlin, Germany; 2 Department of Neurosurgery, University Medical Center Freiburg, Freiburg, Germany; 3 Centre for Clinical Brain Sciences, University of Edinburgh, Edinburgh, United Kingdom; 4 Stroke Division, Florey Institute of Neuroscience and Mental Health, Melbourne, Victoria, Australia; 5 F.M. Kirby Neurobiology Center, Boston Children's Hospital, and Department of Neurology, Harvard Medical School, Boston, United States of America; 6 Center for Stroke Research Berlin, Charité–Universitätsmedizin, Berlin, Germany; 7 German Center for Neurodegenerative Diseases (DZNE) Berlin site, Berlin, Germany; 8 University of Tasmania, School of Medicine, Faculty of Health, Medical Sciences Precinct, Hobart, Tasmania, Australia; 9 Department of Neurology, Spinal Cord Injury Division, The Neurological Institute, The Ohio State University, Wexner Medical Center, Columbus, United States of America; 10 Department of Neuroscience and Center for Brain and Spinal Cord Repair, Department of Physical Medicine and Rehabilitation, The Neurological Institute, The Ohio State University, Wexner Medical Center, Columbus, United States of America; University of California San Francisco, UNITED STATES

## Abstract

Olfactory ensheathing cell (OEC) transplantation is a candidate cellular treatment approach for human spinal cord injury (SCI) due to their unique regenerative potential and autologous origin. The objective of this study was, through a meta-epidemiologic approach, (i) to assess the efficacy of OEC transplantation on locomotor recovery after traumatic experimental SCI and (ii) to estimate the likelihood of reporting bias and/or missing data. A study protocol was finalized before data collection. Embedded into a systematic review and meta-analysis, we conducted a literature research of databases including PubMed, EMBASE, and ISI Web of Science from 1949/01 to 2014/10 with no language restrictions, screened by two independent investigators. Studies were included if they assessed neurobehavioral improvement after traumatic experimental SCI, administrated no combined interventions, and reported the number of animals in the treatment and control group. Individual effect sizes were pooled using a random effects model. Details regarding the study design were extracted and impact of these on locomotor outcome was assessed by meta-regression. Missing data (reporting bias) was determined by Egger regression and Funnel-plotting. The primary study outcome assessed was improvement in locomotor function at the final time point of measurement. We included 49 studies (62 experiments, 1,164 animals) in the final analysis. The overall improvement in locomotor function after OEC transplantation, measured using the Basso, Beattie, and Bresnahan (BBB) score, was 20.3% (95% CI 17.8–29.5). One missing study was imputed by trim and fill analysis, suggesting only slight publication bias and reducing the overall effect to a 19.2% improvement of locomotor activity. Dose-response ratio supports neurobiological plausibility. Studies were assessed using a 9-point item quality score, resulting in a median score of 5 (interquartile range [IQR] 3–5). In conclusion, OEC transplantation exerts considerable beneficial effects on neurobehavioral recovery after traumatic experimental SCI. Publication bias was minimal and affirms the translational potential of efficacy, but safety cannot be adequately assessed. The data justify OECs as a cellular substrate to develop and optimize minimally invasive and safe cellular transplantation paradigms for the lesioned spinal cord embedded into state-of-the-art Phase I/II clinical trial design studies for human SCI.

## Abstract 2

This meta-analysis study examines the effects of transplanting olfactory ensheathing cells in rodents with experimental spinal cord injury, finding evidence for significant recovery and identifying aspects of the procedure that influence the effect size.

## Author Summary

Spinal cord injury converts into a debilitating disease affecting millions of chronic patients worldwide. Despite increased molecular knowledge over the last decades, no causal pharmacological or cellular therapy has proven effective so far. Due to their unique regenerative capabilities and their autologous origin, olfactory ensheathing cells (OECs) constitute an appealing candidate for topical cell transplantation. In contrast to few and heterogeneous experimental reports of OEC transplantation after spinal cord injury in humans, a considerable number of preclinical studies have been conducted applying OEC transplantation in rodent models. We set out to conduct a systematic review and meta-analysis to assess preclinical efficacy of OEC transplantation. We detected a significant overall increase of functional neurological recovery in animals after OEC transplantation compared to the control group. This effect was not distorted by publication bias. We identified several specific hallmarks of the cell transplantation procedure that determine the effect size of the transplantation. Our findings delineate conditions for optimized OEC transplantation into lesioned spinal cords and its relevance for effective translation to human trials.

## Introduction

Cellular transplantation strategies applying different cellular sources have been tested in experimental spinal cord injury (SCI) models as a possible treatment to propagate functional recovery [1–3]. Restoration of function after SCI remains one of the most formidable challenges in regenerative medicine, and the development of effective treatments is an unmet medical need. Pioneering studies by Ramón-Cueto and Nieto-Sampedro [4] reported the purification of olfactory ensheathing cells (OECs) to study regenerative paradigms and were followed by seminal work from the Raisman group, who showed that OEC transplants were populated by host axonal fibers after SCI, associated with neurological recovery [5]. Besides promoting neurite growth, subsequent studies unraveled guidance, neuroprotective, angiogenetic, phagocytic, localized immune modulatory, and remyelination properties as underlying neurobiological effector mechanisms [6–9]. OEC transplantation restored salutatory nerve conduction of sensory axons [10] and fostered the recovery of locomotion after SCI [11–13]. Conceptually, OEC transplantation can be classified as a bridging, non-relay approach [14].

**Fig 1:**
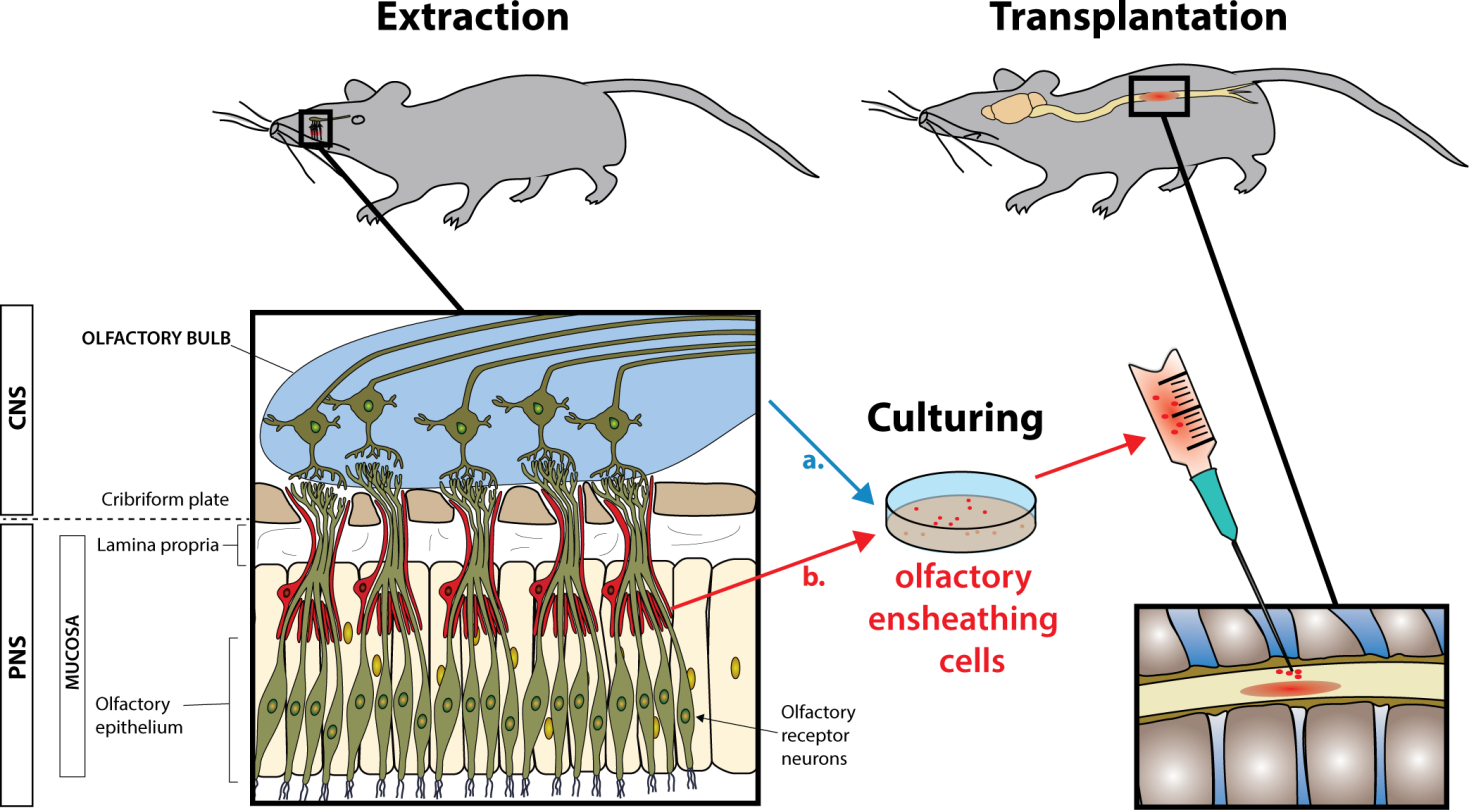
Autologous olfactory ensheathing cell (OEC) transplantation. OECs are extracted from a. the olfactory bulb or b. the olfactory mucosa (left side). After cell propagation, OECs are transplanted on the injured spinal cord (right side). OECs are transplanted either to the lesion core or rostral/caudal parenchymal areas juxtaposed to the lesion site after acute, subacute, or beginning chronic time frames after SCI in varying concentrations and dosages. With their unique ability to reorganise the glial scar and guide regenerating axons from the peripheral to the central nervous system (reviewed recently [6]), OECs are being regarded as a promising cellular approach for SCI treatment. Intramedullary OEC transplantation in proximity to the injured spinal cord site may facilitate regenerating fibres to overcome molecular axonal outgrowth inhibitors surrounding the forming scar and promote functional outcome improvement. There is heterogeneity in morphology, antigen expression, and function in OECs derived from olfactory mucosa versus olfactory bulb [15,16].

The viability as autologous graft and genetic stability of OECs supports their feasibility for clinical translation. OECs comprise a unique and highly specialized cell type, physiologically located at the border between the central nervous system (CNS) and peripheral nervous system (PNS), since the olfactory system is part of both the PNS and CNS (Fig 1).

After olfactory nerve injury, olfactory receptor neurons located in the olfactory epithelium (PNS) can extend axons that enter the olfactory bulb (CNS), thus representing a highly specialized aspect of physiological axonal regeneration in the mammalian CNS [6–9]. This regenerative ability is attributed to a distinct growth-enabling cellular environment composed of OECs, which can be derived from peripheral (mucosal OECs) or central sites (olfactory bulb).

Findings from studies supporting the effect of OEC transplantation were challenged by the landmark “Facilities of Research–Spinal Cord Injury (FOR-SCI) replication” project [17], which confirmed some axonal outgrowth but failed to reproduce an effect on locomotor outcome after OEC transplantation [18]. Importantly, replication alone is an informative tool for deciphering the value for translation. Nevertheless, the probability of replicating “true” findings itself is low (replication power). Mathematically, the chance to obtain a significant result and, hence, “reproduce” data is just about 50% in case of a *p*-value of about 0.05 [19,20].

A related challenge for the predictive value is inflated effect sizes (“Delta inflation” [21]) due to missing data and inappropriate power calculations, which lead to underpowered trials that are unable to confirm or reject a null hypothesis [22]. Omission of negative data renders an objective prioritization of experimental interventions for translation toward clinical trials difficult. The fragile and resource-intense translational path depends on objective measures, and its efficiency will be dependent on a bi-directional dialogue [23]. Meta-analyses based on systematic reviews contribute to this dialogue, as they are able to monitor for missing data and inflated effect sizes [24–26]. First comprehensive approaches to structured appraisal of in vivo evidence for cell-based therapies in SCI have been proposed [27].

Since the discovery of the effect of OECs on propagating neurite growth, various OEC cell preparations (either olfactory mucosa or olfactory bulb derived) have been developed and tested. Given the conflicting results of these experiments, we investigate the in vivo evidence for the efficacy of OEC transplantation after traumatic experimental SCI, applying a systematic review and meta-analysis with the DerSimonian and Laird random effects model. We use meta-regression to determine the influence of different OEC transplantation paradigms on estimates of efficacy. We use Funnel plotting, Egger-regression, and the trim-and-fill method to delineate the occurrence and impact of publication bias. We set out to integrate heterogeneity and missing data as important challenges for clinical translation.

## Results

### Study Selection

**Fig 2:**
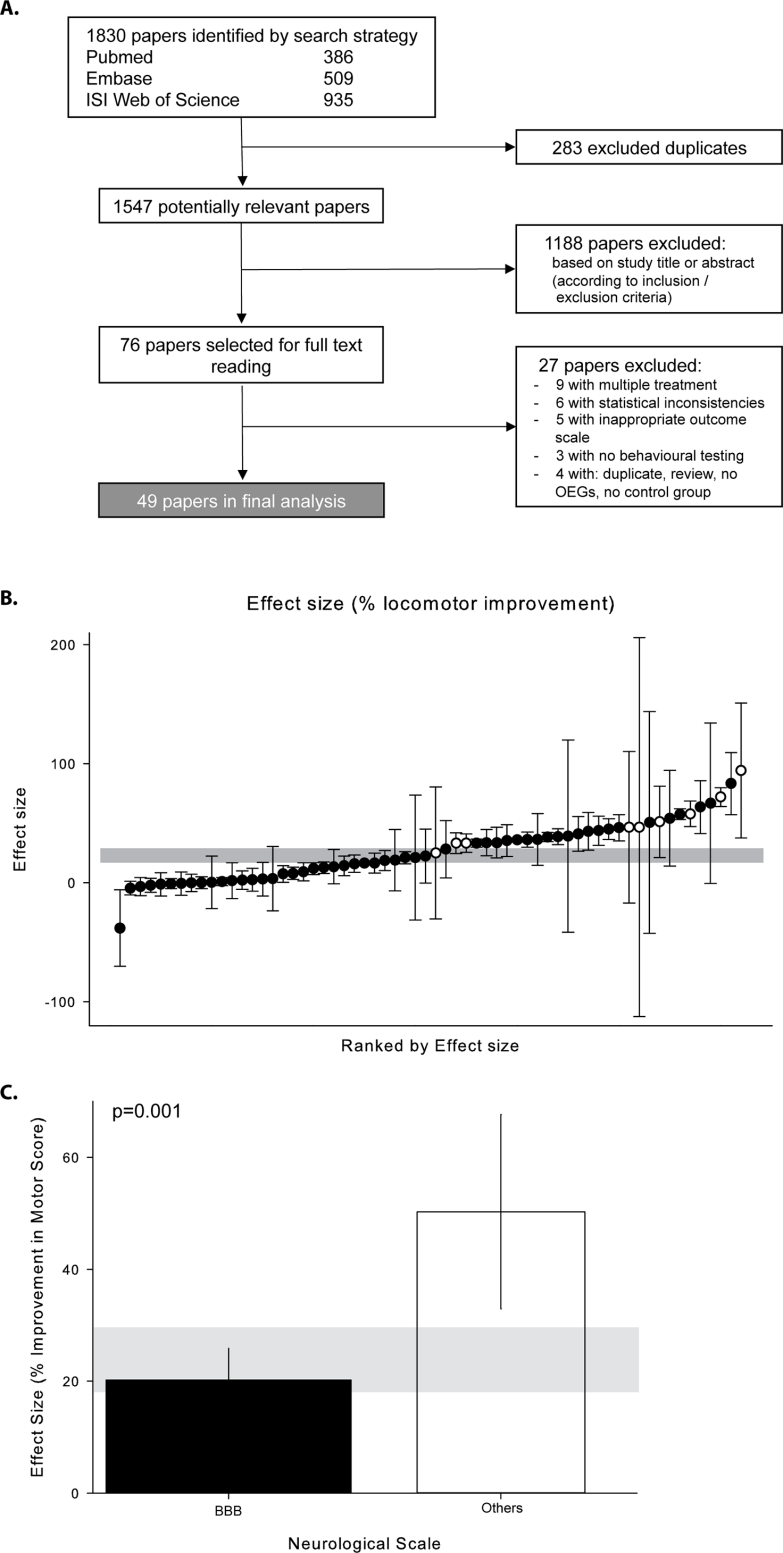
Study selection. (A) Interventional preclinical OEC transplantation studies analyzing effects on locomotor recovery after SCI. (B) Effect size in percent according to rank; black dots represent studies applying the Basso, Beattie, and Bresnahan (BBB) score for neurobehavioral outcome assessment, and white dots indicate studies using other scores. The vertical error bars represent the 95% CI of individual studies, and the horizontal gray bar represents 95% CI of all analyzed studies (overall effect size 23.6 [95% CI 17.8–29.5]). (C) Meta-regression demonstrating the effect size for studies reporting motor outcome by open field BBB testing is comparatively lower when compared with other scores (e.g., Tarlov scale).

The literature search identified 1,830 publications, of which 1,754 were excluded in the first instance (Fig 2A: 283 excluded duplicates, 1,188 excluded based on abstract). Seventy-six studies were selected for further investigation, of which a final number of 49 met the prespecified inclusion criteria. Nine studies were excluded because they reported combined interventions (“cocktails”), three studies did not measure behavioral outcome, five studies had an inappropriate outcome scale, one study was a duplicate, one study was a review, one study did not transplant OECs, a further one did not include a control group, and six studies could not be taken into account due to statistical inconsistencies. Sixty-two experiments reporting outcome in 1,164 animals contributed to our final analysis (Fig 2A).

### Study Details

**Fig 3:**
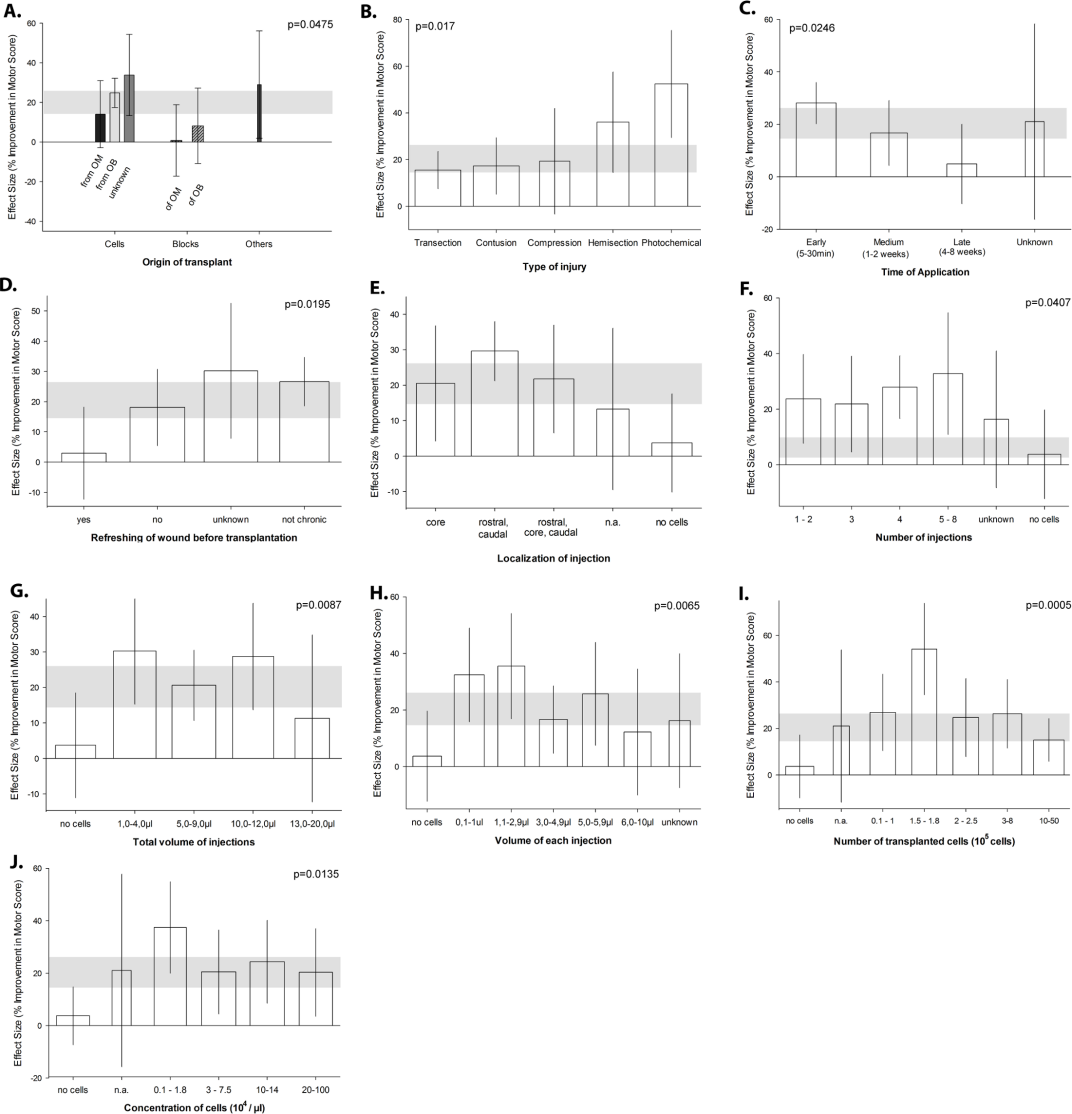
Differential effects of OEC transplantation paradigms on locomotor outcome indicated by the BBB-score. (A) Effect of the origin of transplant (olfactory bulb versus olfactory mucosa derived), cell dispersion (suspension versus tissue block), (B) experimental SCI model, (C) time of OEC application (acute, subacute, beginning chronic lesion milieu), (D) additional surgical scar resection before OEC transplantation, (E) localization of injections (rostral/caudal parenchyma versus lesion core), (F) number of injections per OEC transplantation, (G) total volume of OEC suspension per animal, (H) fractionated volume per injection, (I) number of transplanted cells, and (J) concentration of OEC on the effect size. Meta-regression identifies each aspect of study design significantly associated with different observed efficacies. Vertical error bars represent the 95% CI, and the horizontal gray bar represents the 95% CI of all analyzed studies. The width of the columns depicts the log of the number of used animals in this group.

**Fig 4:**
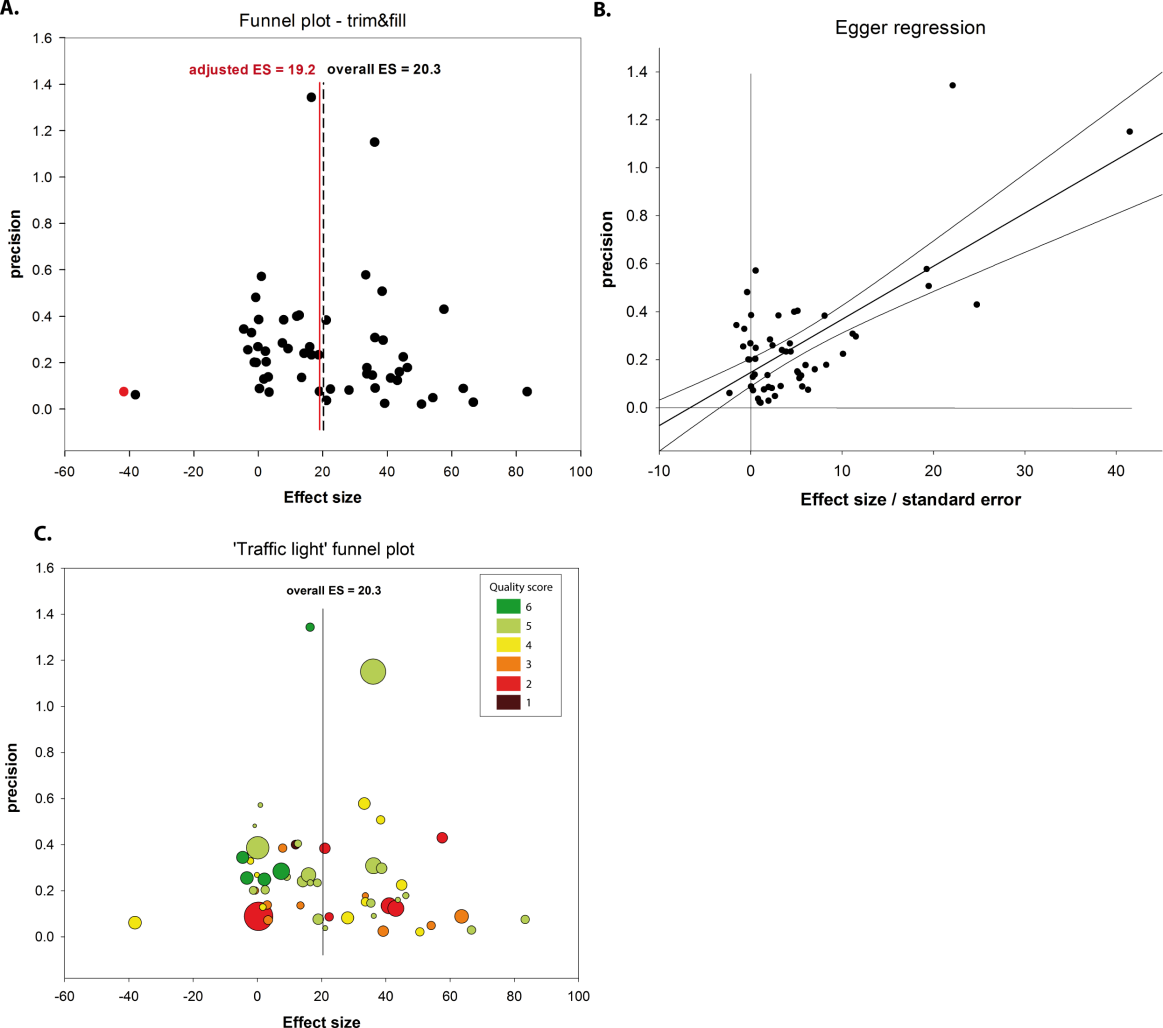
Assessment of publication bias. (A) Funnel plot displays the precision plotted against the effect size. When considering the Funnel plot without any imputation from the trim and fill method (black dots only), the analysed 62 experiments indicate an overall effect size (ES) of 20.3%. When applying the trim and fill method to detect possible missing experiments based on the funnel plot’s asymmetry, the overall effect size results in a reduced effect size of 19.2%. One imputation was done, indicating a possibly missing experiment (red dot). (B) Egger regression illustrates the precision (one divided by the standard error of the mean) plotted against effect size divided by the standard error of the mean. Egger regression line does not intersect the origin, indicating possibly underlying publication bias. (C) “Traffic light” colored funnel plot adds two layers of information in the context of study quality. The circle sizes represent the number of animals in the individual experiment and the dot colors reflect the individual points on the quality score. The “traffic light” funnel plot demonstrates that studies with higher quality scores (≥5; green dots) relate closer to the corrected calculated effect size, although the stratification did not account for significance heterogeneity (Quality score ≥5: 18.7 [95%CI 10.6–26.7]; <5: 22.1 [10.3–33.9]). However, the “traffic light” funnel plot also identified a cluster of studies reporting high study quality likely to further correct the observed effect size below the adjusted effect size of the trim-and-filled funnel plot.

All studies were done in rats, and all OEC transplantations were applied to the intradural compartment. The animals were subject to five different modalities of experimental SCI; 17 studies used transection (23 experiments), 17 studies used contusion (20 experiments), six used hemisection (seven experiments), six used photochemical injury (six experiments), and four used compression (six experiments). All experiments lesioned the spinal cord at thoracic levels T8-13. OECs were either transplanted immediately after injury (32 experiments) or delayed (30 experiments), the longest post-SCI interval being 270 days. Most of the experiments used the Basso, Beattie, and Bresnahan [BBB] score [16] for evaluation of functional recovery (53 experiments). The BBB score is a 21-point open field locomotor rating score that categorizes stepping, paw placement, fore- and hindlimb coordination, tail position, joint movement, hindlimb movement, trunk position, and stability [16]. The overall analysis (Fig 2B) revealed a distinct difference in reported outcome using the BBB score compared to other scores, including directed forepaw reaching (three experiments), kinematic analysis (one experiment), beam walking (one experiment), horizontal rope walking (one experiment), tape removal (one experiment), tape sensing (one experiment), and spontaneous vertical exploration (one experiment) (BBB: effect size [ES] 20.2%; others: ES 50.3%) (Fig 2C). We therefore excluded studies in which scores other than the BBB-score have been assessed exclusively from the subsequent analysis (Figs 3 and 4).

### Neurobehavioral Outcome

Overall, OEC transplantation after traumatic SCI improved locomotor recovery by 23.6% (95% CI 17.8–29.5; I^2^ = 96.4%, 62 experiments, 1,164 animals) when including various test paradigms (Fig 2B). In experiments reporting BBB measures only, the effect was 20.3% (95% CI 14.4–26.1; I^2^ = 96.7%, 53 experiments, 1,027 animals). Meta-regression showed several aspects of study design that were significantly associated with different observed efficacies:

- Origin of transplant: Transplantation of OECs as single cell suspension showed better results than use of tissue blocks. Both cells and blocks originating from the olfactory bulb induced higher rates of recovery than those from olfactory mucosa (R^2^ = 16.6%, Fig 3A)
- Method of injury: The most severe SCI paradigm in terms of functional neurological recovery was a complete transection, followed by contusion injury and hemisection. Neurological recovery is most extensive after photochemical injury compared to other types of SCI (R^2^ = 14.0%, Fig 3B).
- Timing of transplantation: OEC transplantation derived from mucosa or olfactory bulb immediately after injury (hyperacute phase, 5–30 min) was associated with greater efficacy than subacute (1–2 wk) or chronic time windows (4–8 wk) after SCI (R^2^ = 14.5%, Fig 3C).
- Surgical resection of fibrotic scar (“refreshing” of wound): Microsurgical resection of fibrotic scar tissue from the lesion site was associated with a reduced degree of neurological recovery. In particular, in the chronic phase, surgical resection after a scar has been formed was detrimental, whereas manipulations during the acute phase appear less detrimental (R^2^ = 16.4%, Fig 3D).
- Transplantation region: Injections restricted to the core of the spinal cord lesion led to the worst results compared to injections to the caudal and rostral parenchyma juxtaposed to the lesion rim. Additional OEC transplantation to the lesion core concomitant to rostral and caudal injection had no significant effect on effect size. When cells were not dissociated (e.g., blocks of OEC), only very low effect sizes were observed (R^2^ = 22.4%, Fig 3E).
- Number of injections: More widespread distribution of the transplantation volume by multiple injection spots was associated with larger effect sizes compared to singular injections (R^2^ = 18.0%, Fig 3F).
- Total transplantation volume: Injections into the rat spinal cord up to the volume of 12 μl are associated with robust comparable effect sizes, whereas larger volumes resulted in reduced effects. By contrast, when cells were not invasively anchored to the lesioned cord (no cell injections), the efficacy was very low (R^2^ = 22.8%, Fig 3G).
- Volume of each injection: Singular injection of smaller volumes up to 2.9 μl are associated with better outcomes compared to larger injection volumes (R^2^ = 29.9%, Fig 3H).
- Dosing—cell numbers: Escalation of cell numbers result in a dose-response-curve of efficacy. While being associated with stable effect sizes in the range between 10,000 and 100,000 cells, reaching a maximum in the range for 150,000–180,000 cells, the transplantation of more than 200,000 OECs started to be associated with decreased effect size (R^2^ = 34.9%, Fig 3I), likely indicating increasingly overlapping neurotoxic effects of high dose cellular transplants requiring higher injection volumes (see also Fig 3J).
- Concentration of cells: The most efficient applied cell concentration was 1,000–18,000 cells per microliter. Higher concentrations showed lower effect sizes (R^2^ = 20.1%, Fig 3J)

### Study Quality and Publication Bias

The median score in the quality checklist was 5 (IQR 3–5), relating to a maximum score value of 9. All studies were published in peer-reviewed journals, in which 28 studies reported blinded assessment of outcome and 25 did not. Only 17 studies (32%) out of 53 reported random allocation to treatment or control group. None of the studies made use of sample size calculation, and only one used blinded induction of injury. None of the single study quality items accounted for a significant amount of heterogeneity.

The combined 9-point item quality score revealed a trend for the relationship between effect size and study quality, but showed no statistical significance. Studies with lower-quality score items correspond with higher effect sizes, whereas higher quality studies associate with lower effect size (e.g., quality score 5: 22.5% [95%CI 13.6–31.3], quality score 2: 32.3% [95%CI 13.1–51.5]).

We assessed the dataset for missing data by applying funnel plotting and Egger regression analysis (Fig 4). Publication bias is a major source of missing data and attributed to unpublished studies mostly reporting negative results. Orthodox preclinical systematic review cannot assess this dilemma properly, as only published results are included, leading consequently to the risk of inflated effect sizes (so-called file drawer problem).

Trim and fill analysis was used to identify theoretically missing experiments based on the funnel plot’s symmetry. Only one missing experiment was imputed, suggesting that present publication bias for this cohort was only minor, adjusting the effect size only marginally from 20.3% to 19.2% (Fig 4A). The finding of modest publication bias was confirmed by Egger regression (Fig 4B).

Although there is reason to believe that studies at risk of bias might give overstatements of efficacy, it is also plausible that such studies may give less precise estimates. Therefore, in a post hoc analysis, we investigated whether studies with a higher quality score (i) were closer to the overall calculated effect size or (ii) had higher precision (1/SE) (Fig 4B). The modified “traffic light” funnel plot implies that studies with higher-quality scores are closer to the overall corrected calculated effect size than studies of lower quality. Moreover, high-quality studies (≥5) appeared to be associated with a higher (averaged) precision compared with lower-quality studies (<5). Together, the “traffic light” funnel plot provides a visual tool to identify study clusters of high quality and precision that may indicate the true effect size is below the adjusted effect size of the trim-and-filled funnel plot.

## Discussion

This systematic review and meta-analysis of preclinical animal literature identified 49 studies of olfactory ensheathing cell transplantation reporting 23.6% improvement of functional outcome. Data from 1,164 animals comprising 62 experiments were stratified for neurobiological and study characteristics, which accounted for significant proportion of in-between study heterogeneity. Motor-score sub-analysis was confined to studies including assessments of neurobehavioral recovery applying the most commonly used BBB-score characterized by smaller effect sizes (20.3%) compared to other measures of motor recovery (Tarlov scale and others). Statistical techniques to detect and correct for publication bias revealed a slight overestimation of effect sizes due to theoretically missing experiments resulting in a corrected estimated efficacy of 19.2%. Besides minimizing variability to enable the most accurate reproduction (e.g., FORESCI Initiative [17]), an alternative approach is the deliberate incorporation of heterogeneity in multiple modeling characteristics in order to evaluate the impact of study differences with regards to the effect size.

Bulb- and mucosa-derived OECs differ substantially, for example, with regards to their proliferative ability over time [28,29]. OECs derived from the olfactory bulb provided a larger effect on locomotor recovery compared with OECs derived from mucosa. The transplantation of OEC as purified and cultured cells reported better outcome compared to animals receiving blocks of olfactory tissue, which are less likely to integrate. Similar effects after clinically relevant contusion, compression, and transection SCI support the robustness, irrespective of model differences, such as axons being fully cut or just severed or the amount of bleeding due to injured arteries and veins. The purity of the OEC transplants is most commonly determined by immunocytochemical markers (p75NTR, S100, GFAP), but there is no stringent standard for the OEC preparation [28,30]. Of note, we observed no significant impact of the purity assessment with regards to the elicited locomotor recovery, but obvious trends. It is assumed that all OEC cultures may contain a small proportion of Schwann cells (e.g., trigeminal derived) until proven otherwise or other critical cells able to augment anatomical and functional recovery after SCI [30]. Immediate OEC transplantation within 30 min after injury was superior to subacute (1–2 wk) and delayed application (4–8 wk), indicative of a neuroprotective effect linked to a distinct therapeutic time window, which might be different in humans. Surprisingly, the benefits of early transplantation were not overruled by an anticipated elevated neurotoxic risk due to injection into oedematous tissue, which might amplify the extracellular pressure imposed on spared spinal neurons and axons after SCI. The invasive removal of distal axonal stumps and formed scar tissue (“wound refreshing”) resulted in worsened effect sizes, supporting the functional relevance of scar-associated neuronal circuitry either being spared or formed de novo after SCI [31]. In addition, the inherent ability of OECs to reorganize the glial scar (expression of growth promoting matrix molecules and proteolytic enzymes) might render surgical scar resection unnecessary.

Despite migratory properties and the ability to intermingle with host scar-forming cells, the localization of transplantation matters. Injection to rostral and caudal parenchyma juxtaposed to the lesion site is significantly more effective than to the lesion core. This is in line with the proposed underlying neurobiological mechanisms such as neuroprotection and the propagation of neuroplasticity and remyelination, which do not apply to the pan-necrotic lesion core. Multiple injections with smaller volumes are associated with elevated locomotor effects compared to a reduced number of injections, which in turn require larger cell numbers and injection volumes. Considering technical aspects of transplantation, it appears that neurotoxicity generated by injection into the spinal cord itself is comparatively low (likely as long as no vessel is affected) and depends largely on the amount of volume being injected. The results favour multiple injections over large cell deposits through singular injection (declining benefit-risk ratio with increasing injection volume). The data also suggest that the toxicity of invasive transplantation is largely determined by the injected volume and the attributable hydrodynamic dissection pressure [32]. In brief, (i) a volume over 3 μl per injection, (ii) a total transplantation volume exceeding 12 μl, and (iii) an OEC concentration higher than 180,000 cells/μl are associated with reduced locomotor outcome. The dose–response relationship between the amount of transplanted OECs and effect size confirms the biological plausibility. With dose escalation above 180,000 cells, increasingly neurotoxic effects start to predominate, with a ceiling to the beneficial effect likely due to elevated injection volumes (hydrodynamic dissection pressure). This ceiling effect is further aggravated by a limited engraftment of cells per tissue unit [33]. In terms of efficacy, autologous OECs did not differ from xenogenous sources. The parameters obtained here from animals provide a framework for scaling for spinal transplantation for translation into paradigms of human SCI.

A recent meta-analysis summarized 10 treatment series reporting on 1,193 chronic SCI patients and demonstrated an improvement rate of 39% on the American Spinal Injury Association (ASIA) impairment scale after chronic SCI [34] and OEC transplantation. However, it is noteworthy that all publications had low methodological quality and were lacking appropriate control groups. The mortality associated with full anesthesia and surgery was 0.35% (*n* = 2 out of 566). Reported adverse effects included fever, mild anemia, syringomyelia, cerebrospinal fluid leakage, aseptic meningitis, and sensory and motor deterioration. The safety being claimed in some reviews summarizing experimental OEC mode of action was challenged by a recent clinical case report identifying an intramedullar tumor formation 8 y after spinal mucosal OEC transplantation presenting with progressive pain [35]. The tumor required surgical resection and was identified as being composed of respiratory epithelium, submucosal glands with goblet cells, and mucosal mass. The risk of delayed tumor formation points toward a low ability to attack and remove transplanted cells of ectopic origin. Given the mortality attributable to anaesthesia and surgery per se, complications due to the transplantation and the risk of delayed tumor formation even when applying autologous cells render the long-term safety in human SCI uncertain and needs to be integrated in a benefit–risk ratio calculation. Experimental OEC SCI transplantations investigated here do not cover corresponding long-term observational time windows.

The following limitations apply to this study. Our analysis depends on the validity of locomotor recovery scales used, which has been questioned [36]. However, the applied outcome measures scales are in widespread use and are considered the most informative, commonly used neurobehavioral element to assess outcome in modelling SCI. For this reason, it is unlikely that a preclinical intervention would be approved for translation if it did not result in improvements in the BBB score. Furthermore, all studies reported exclusively rat models of SCI. Large animal models are of translational relevance but are limited as being frequently underpowered and applying widely heterogeneous outcome measures and could therefore not be included. The funnel plot test is only one way to visualize missing data and can be debated [37]. We therefore included the individual experimental quality and animal number in the funnel plot, leading to a “traffic light” modification. In summary, systematic reviews and meta-analysis provide a rather broad estimate of efficacy.

## Conclusion

Due to the unique properties of fostering a plethora of neurobiological repair mechanisms including axon sprouting, myelination, and neuroprotection, autologous OECs are considered a candidate cellular source for transplantation into the lesioned spinal cord. This systematic meta-analysis reports a substantial overall effect of OEC transplantation after experimental SCI. The data suggests more effective OEC transplantation paradigms, (i) when being derived from the olfactory bulb compared with mucosa-derived OECs, (ii) when injected to rostral-caudal parenchyma compared with the injury epicenter, and (iii) when being fractionated, allowing for smaller injection volumes. Invasive surgical resection of the fibrotic scar at the lesion site should be avoided. Publication bias was minimal and affirms the translational potential in terms of preclinical efficacy. Safety issues cannot be addressed here sufficiently, given that rodent models rarely study long-term follow-up. Therefore, late complications in humans cannot be addressed sufficiently. At present, the ideal cell for transplant-mediated CNS repair has not been identified. Based on validated efficacy, OECs qualify as autologous cell source to elucidate and optimize cell invasive transplantation paradigms after human SCI as a component for escalated treatment concepts, e.g., by applying phase II trials with informative secondary endpoints aiming to characterize treatment responders and patients prone to develop transplantation-associated complications.

## Materials and Methods

The study protocol was finalized in advance of any data collection and is accessible online (http://www.dcn.ed.ac.uk/camarades/files/PROSPERO%20Protocoll%20OEG.pdf). The methodology and statistical approach is described in greater detail elsewhere [38].

### Search Strategy

To identify animal studies describing the effect of OEC transplantation on neurobehavioral recovery after traumatic experimental SCI, the following search terms were used for PubMed, EMBASE, and ISI Web of Science (search conducted October 2, 2014): (olfactory OR olfactory bulb OR olfactory lamina propria OR olfactory ensheathing cells) AND (spinal cord injury OR hemisection OR contusion injury OR dorsal column injury OR complete transection OR corticospinal tract injury). Search results were limited to animals. The animal filter was modified for the search in PubMed [39]. Two investigators (R.W. and J.R.) independently assessed the search results.

### Inclusion and Exclusion Criteria

Studies were included if they reported the effects of OEC transplantation in animal models after spinal cord injuries, including contusion, compression, hemisection, transection, and photochemical injury. We included SCI experiments comparing functional outcome in a group of animals receiving OEC treatment with a control group receiving no treatment or vehicle treatment. Non-traumatic models of SCI were excluded, as well as studies reporting only combined treatments. For studies to be included they had to report the number of animals for each group, the mean effect size, and its standard deviation or standard error of the mean.

### Data Extraction

We extracted details of individual study characteristics from each publication, and when a single publication reported more than one experiment, these data were extracted and treated as independent experiments. Study characteristics were extracted, including the gender and breed of the animals, time route and dose of transplantation, anaesthetic, and method of injury, as well as adjuvant treatment. Functional outcome was assessed for each experiment. Where data were expressed in text, numerical values were extracted. Where the outcome was expressed graphically only, Universal Desktop Ruler (Version 3.6, AVPsoft) was used to visually extract the data points. Only the final time point of the assessment of functional recovery was included.

### Quality Assessment

As an assessment for “risk of bias,” we assessed the methodological quality of each study using a modified 9-point item quality checklist, adapted from the CAMARADES (Collaborative Approach to Meta Analysis and Review of Animal Data from Experimental Studies) quality checklist [40]: (i) reporting of a sample size calculation, (ii) control of animals’ temperature, (iii) use of anaesthetics other than ketamine (because of its marked intrinsic neuroprotective activity), (iv) randomized treatment allocation, (v) treatment allocation concealment, (vi) blinded assessment of outcome, (vii) publication in a peer reviewed journal, (viii) statement of compliance with regulatory requirements, and (ix) statement of potential conflicts of interest. This quality checklist overlaps substantially with the ARRIVE (Animal Research: Reporting In Vivo Experiments) guideline for reporting animal research [41].

### Analysis

A normalized effect size (ES) for each comparison was calculated, defined as the improvement of outcome in the treatment group compared with that in the control group with reference to the outcome of an untreated, unlesioned, “sham” animal. The attributed size of the control group was adjusted if a single control group was compared to more than one treatment group. We used DerSimonian and Laird random effects model meta-regression to calculate an overall estimate of effect size. The analysis was stratified according to the method of injury, type of treatment, time of application, number of transplanted cells, quality assessment score, adjuvant treatment, time of assessment, medium used for cell culture, transplant origin, assessment of OEC purity, details of surgical procedure, and type of anaesthetic. Meta-regression was used to determine how much heterogeneity can be explained by study design characteristics. Random effects meta-regression was conducted by taking into account both within-study and between-study variance using STATA13 with a significance level of *p* < 0.05. In the regression model, the variance in the dependent variable that is accounted for by covariates is used to calculate an adjusted R^2^, a measure of how much residual heterogeneity is explained by the model [38]. Figures were drawn using SigmaPlot (Systat Software Inc, Version 12).

## Supporting Information

S1 Checklist PRISMA checklist. (DOC)

S1 Data Primary raw data (Figs 2B–4C). (XLSX)

S1 Table Details of included studies. (DOCX)

S1 Text Formula used to calculate effect sizes. (DOCX)

## Abbreviations

ASIA: American Spinal Injury Association
CNS: central nervous system
ES: effect size
FOR-SCI: Facilities of Research–Spinal Cord Injury
IQR: interquartile range
OEC: olfactory ensheathing cells
PNS: peripheral nervous system
SCI: spinal cord injury

## References

[1] Ruff CA, Wilcox JT, Fehlings MG. Cell-based transplantation strategies to promote plasticity following spinal cord injury. Experimental neurology. 2012;235(1):78–90. 10.1016/j.expneurol.2011.02.010 . 21333647

[2] Tetzlaff W, Okon EB, Karimi-Abdolrezaee S, Hill CE, Sparling JS, Plemel JR, et al A systematic review of cellular transplantation therapies for spinal cord injury. Journal of neurotrauma. 2011;28(8):1611–82. 10.1089/neu.2009.1177 ; PubMed Central PMCID: PMCPmc3143488. 20146557 PMC3143488

[3] Thuret S, Moon LD, Gage FH. Therapeutic interventions after spinal cord injury. Nature reviews Neuroscience. 2006;7(8):628–43. 10.1038/nrn1955 . 16858391

[4] Ramon-Cueto A, Nieto-Sampedro M. Regeneration into the spinal cord of transected dorsal root axons is promoted by ensheathing glia transplants. Experimental neurology. 1994;127(2):232–44. 10.1006/exnr.1994.1099 . 8033963

[5] Li Y, Field PM, Raisman G. Repair of adult rat corticospinal tract by transplants of olfactory ensheathing cells. Science (New York, NY). 1997;277(5334):2000–2. . 9302296 10.1126/science.277.5334.2000

[6] Roet KC, Verhaagen J. Understanding the neural repair-promoting properties of olfactory ensheathing cells. Experimental neurology. 2014;261c:594–609. 10.1016/j.expneurol.2014.05.007 . 24842489

[7] Raisman G, Li Y. Repair of neural pathways by olfactory ensheathing cells. Nature reviews Neuroscience. 2007;8(4):312–9. 10.1038/nrn2099 . 17342173

[8] Barnett SC, Riddell JS. Olfactory ensheathing cell transplantation as a strategy for spinal cord repair—what can it achieve? Nature clinical practice Neurology. 2007;3(3):152–61. 10.1038/ncpneuro0447 . 17342191

[9] Richter MW, Roskams AJ. Olfactory ensheathing cell transplantation following spinal cord injury: hype or hope? Experimental neurology. 2008;209(2):353–67. 10.1016/j.expneurol.2007.06.011 . 17643431

[10] Toft A, Scott DT, Barnett SC, Riddell JS. Electrophysiological evidence that olfactory cell transplants improve function after spinal cord injury. Brain: a journal of neurology. 2007;130(Pt 4):970–84. 10.1093/brain/awm040 . 17438017

[11] Lu J, Feron F, Mackay-Sim A, Waite PM. Olfactory ensheathing cells promote locomotor recovery after delayed transplantation into transected spinal cord. Brain: a journal of neurology. 2002;125(Pt 1):14–21. . 11834589 10.1093/brain/awf014

[12] Lopez-Vales R, Fores J, Navarro X, Verdu E. Chronic transplantation of olfactory ensheathing cells promotes partial recovery after complete spinal cord transection in the rat. Glia. 2007;55(3):303–11. 10.1002/glia.20457 . 17096411

[13] Ziegler MD, Hsu D, Takeoka A, Zhong H, Ramon-Cueto A, Phelps PE, et al Further evidence of olfactory ensheathing glia facilitating axonal regeneration after a complete spinal cord transection. Experimental neurology. 2011;229(1):109–19. 10.1016/j.expneurol.2011.01.007 ; PubMed Central PMCID: PMCPmc3085566. 21272578 PMC3085566

[14] Bregman BS, Kunkel-Bagden E, Reier PJ, Dai HN, McAtee M, Gao D. Recovery of function after spinal cord injury: mechanisms underlying transplant-mediated recovery of function differ after spinal cord injury in newborn and adult rats. Experimental neurology. 1993;123(1):3–16. 10.1006/exnr.1993.1136 . 8405277

[15] Higgins JP, Thompson SG, Deeks JJ, Altman DG. Measuring inconsistency in meta-analyses. BMJ. 2003;327(7414):557–60. 10.1136/bmj.327.7414.557 12958120 PMC192859

[16] Basso DM, Beattie MS, Bresnahan JC. A sensitive and reliable locomotor rating scale for open field testing in rats. Journal of neurotrauma. 1995;12(1):1–21. . 7783230 10.1089/neu.1995.12.1

[17] Steward O, Popovich PG, Dietrich WD, Kleitman N. Replication and reproducibility in spinal cord injury research. Experimental neurology. 2012;233(2):597–605. 10.1016/j.expneurol.2011.06.017 . 22078756

[18] Steward O, Sharp K, Selvan G, Hadden A, Hofstadter M, Au E, et al A re-assessment of the consequences of delayed transplantation of olfactory lamina propria following complete spinal cord transection in rats. Experimental neurology. 2006;198(2):483–99. 10.1016/j.expneurol.2005.12.034 . 16494866

[19] Goodman SN. A comment on replication, p-values and evidence. Statistics in medicine. 1992;11(7):875–9. . 1604067 10.1002/sim.4780110705

[20] Gorroochurn P, Hodge SE, Heiman GA, Durner M, Greenberg DA. Non-replication of association studies: "pseudo-failures" to replicate? Genetics in medicine: official journal of the American College of Medical Genetics. 2007;9(6):325–31. doi: 10.1097GIM.0b013e3180676d79 . 17575498 10.1097/gim.0b013e3180676d79

[21] Aberegg SK, Richards DR, O'Brien JM. Delta inflation: a bias in the design of randomized controlled trials in critical care medicine. Crit Care. 2010;14(2):R77 10.1186/cc8990 20429873 PMC2887200

[22] Howells DW, Sena ES, Macleod MR. Bringing rigour to translational medicine. Nat Rev Neurol. 2014;10(1):37–43. 10.1038/nrneurol.2013.232 . 24247324

[23] Curt A. The translational dialogue in spinal cord injury research. Spinal cord. 2012;50(5):352–7. 10.1038/sc.2011.113 . 22064661

[24] Antonic A, Sena ES, Lees JS, Wills TE, Skeers P, Batchelor PE, et al Stem cell transplantation in traumatic spinal cord injury: a systematic review and meta-analysis of animal studies. PLoS Biol. 2013;11(12):e1001738 10.1371/journal.pbio.1001738 ; PubMed Central PMCID: PMCPmc3866091. 24358022 PMC3866091

[25] Sena ES, Currie GL, McCann SK, Macleod MR, Howells DW. Systematic reviews and meta-analysis of preclinical studies: why perform them and how to appraise them critically. Journal of cerebral blood flow and metabolism: official journal of the International Society of Cerebral Blood Flow and Metabolism. 2014;34(5):737–42. 10.1038/jcbfm.2014.28 ; PubMed Central PMCID: PMCPmc4013765. 24549183 PMC4013765

[26] Watzlawick R, Sena ES, Dirnagl U, Brommer B, Kopp MA, Macleod MR, et al Effect and reporting bias of RhoA/ROCK-blockade intervention on locomotor recovery after spinal cord injury: a systematic review and meta-analysis. JAMA neurology. 2014;71(1):91–9. 10.1001/jamaneurol.2013.4684 . 24297045

[27] Kwon BK, Okon EB, Tsai E, Beattie MS, Bresnahan JC, Magnuson DK, et al A grading system to evaluate objectively the strength of pre-clinical data of acute neuroprotective therapies for clinical translation in spinal cord injury. Journal of neurotrauma. 2011;28(8):1525–43. 10.1089/neu.2010.1296 ; PubMed Central PMCID: PMCPmc3143387. 20507235 PMC3143387

[28] Jani HR, Raisman G. Ensheathing cell cultures from the olfactory bulb and mucosa. Glia. 2004;47(2):130–7. 10.1002/glia.20038 . 15185392

[29] Mayeur A, Duclos C, Honore A, Gauberti M, Drouot L, do Rego JC, et al Potential of olfactory ensheathing cells from different sources for spinal cord repair. PLoS ONE. 2013;8(4):e62860 Epub 2013/05/03. 10.1371/journal.pone.0062860 ; PubMed Central PMCID: PMCPmc3634744. 23638158 PMC3634744

[30] Mackay-Sim A, St John JA. Olfactory ensheathing cells from the nose: clinical application in human spinal cord injuries. Experimental neurology. 2011;229(1):174–80. 10.1016/j.expneurol.2010.08.025 . 20832402

[31] Radojicic M, Reier PJ, Steward O, Keirstead HS. Septations in chronic spinal cord injury cavities contain axons. Experimental neurology. 2005;196(2):339–41. 10.1016/j.expneurol.2005.08.009 . 16153640

[32] Guest J, Benavides F, Padgett K, Mendez E, Tovar D. Technical aspects of spinal cord injections for cell transplantation. Clinical and translational considerations. Brain research bulletin. 2011;84(4–5):267–79. Epub 2010/11/20. 10.1016/j.brainresbull.2010.11.007 . 21087657

[33] Piltti KM, Avakian SN, Funes GM, Hu A, Uchida N, Anderson AJ, et al Transplantation dose alters the dynamics of human neural stem cell engraftment, proliferation and migration after spinal cord injury. Stem cell research. 2015;15(2):341–53. 10.1016/j.scr.2015.07.001 ; PubMed Central PMCID: PMCPmc4600655. 26298025 PMC4600655

[34] Li L, Adnan H, Xu B, Wang J, Wang C, Li F, et al Effects of transplantation of olfactory ensheathing cells in chronic spinal cord injury: a systematic review and meta-analysis. European spine journal: official publication of the European Spine Society, the European Spinal Deformity Society, and the European Section of the Cervical Spine Research Society. 2015;24(5):919–30. 10.1007/s00586-014-3416-6 . 25001890

[35] Dlouhy BJ, Awe O, Rao RC, Kirby PA, Hitchon PW. Autograft-derived spinal cord mass following olfactory mucosal cell transplantation in a spinal cord injury patient: Case report. Journal of neurosurgery Spine. 2014;21(4):618–22. 10.3171/2014.5.spine13992 . 25002238

[36] Ferguson AR, Hook MA, Garcia G, Bresnahan JC, Beattie MS, Grau JW. A simple post hoc transformation that improves the metric properties of the BBB scale for rats with moderate to severe spinal cord injury. Journal of neurotrauma. 2004;21(11):1601–13. 10.1089/neu.2004.21.1601 . 15684652

[37] Simonsohn U, Nelson LD, Simmons JP. p-Curve and Effect Size: Correcting for Publication Bias Using Only Significant Results. Perspect Psychol Sci. 2014;9(6):666–81. 10.1177/1745691614553988 . 26186117

[38] Vesterinen HM, Sena ES, Egan KJ, Hirst TC, Churolov L, Currie GL, et al Meta-analysis of data from animal studies: A practical guide. Journal of neuroscience methods. 2014;221:92–102. 10.1016/j.jneumeth.2013.09.010 . 24099992

[39] Leenaars M, Hooijmans CR, van Veggel N, ter Riet G, Leeflang M, Hooft L, et al A step-by-step guide to systematically identify all relevant animal studies. Laboratory animals. 2012;46(1):24–31. 10.1258/la.2011.011087 ; PubMed Central PMCID: PMCPmc3265183. 22037056 PMC3265183

[40] Macleod MR, O'Collins T, Howells DW, Donnan GA. Pooling of animal experimental data reveals influence of study design and publication bias. Stroke; a journal of cerebral circulation. 2004;35(5):1203–8. 10.1161/01.str.0000125719.25853.20 . 15060322

[41] Kilkenny C, Browne WJ, Cuthill IC, Emerson M, Altman DG. Improving bioscience research reporting: the ARRIVE guidelines for reporting animal research. PLoS Biol. 2010;8(6):e1000412 Epub 2010/07/09. 10.1371/journal.pbio.1000412 ; PubMed Central PMCID: PMCPmc2893951. 20613859 PMC2893951

