# Supplementary material for: Olfactory Ensheathing Cell Transplantation in Experimental Spinal Cord Injury: Effect size and Reporting Bias of 62 Experimental Treatments: A Systematic Review and Meta-Analysis
Source: PLoS Biol. 2016 May 31;14(5):e1002468. doi: 10.1371/journal.pbio.1002468 (PMC4886956; doi:10.1371/journal.pbio.1002468)
Supplement: S1 Table — (DOCX) [file pbio.1002468.s003.docx]

**S1 Table**

| **Study** | **Drug** | **Score used** | **No. of animals** | **Culture medium** | **Lesion level** | **Dose** | **Timepoint of administration** | **Time of assessment (days)** | **Cotreatment** | **Anaesthetic** | **Type of injury** |
| --- | --- | --- | --- | --- | --- | --- | --- | --- | --- | --- | --- |
| Amemori, T (1) | OECs cultured | BBB | 27 | Growth Factors | T8 | 300000 cells | 7d | 63 | cyclosporine, ampicillin, gentamicin sulfate | Volatile | Compression |
| Aoki, M (2) | OM | BBB | 31 | not cultured | T10 | unknown | 14d | 70 | flomoxef | Pentobarbital | Transection |
| Cao, L (3) | OECs cultured | BBB | 23 | Growth Factors | T8 | 200000 cells | 5 min | 56 | cefalozin sodium | Pentobarbital | Transection |
| Carillo-Ruiz, J (4) | whole OB | BBB | 10 | not cultured | T8 | 2 pieces | 5 min | 42 | none | Chloral Hydrate | Contusion |
| Centenaro, L (5) | OLP | BBB | 23 24 24 | not cultured | T8/9 | unknown | 5 min 14d 28d | 80 110 95 | dimorph, baytril | Pentobarbital Volatile Pentobarbital | Transection |
| Chen, H (6) | OECs cultured | BBB | 16 | unknown | T12 | unknown | 28d | 88 | none | Chloral Hydrate | Transection |
| Collazos-Castro, J (7) | OECs cultured | Kinematic analysis | 24 | Antibiotics+ Growth Factors | C7 | 1000000 cells | 5 min | 84 | none | Pentobarbital | Contusion |
| Deng, YB (8) | OECs cultured | BBB | 30 | unknown | T10 | 250000 cells | 30 min | 35 | cyclosporine A, cefalozin | Chloral Hydrate | Contusion |
| García-Alías, G (9) | OECs cultured | BBB | 16 | Growth Factors | T8 | 180000 cells | 30 min | 90 | none | Pentobarbital | Photochemical |
| García-Alías, G (10) | OECs cultured | BBB | 16 | unknown | T8 | 180000 cells | 5 min | 90 | none | Pentobarbital | Photochemical |
| Gorrie, CA (11) | OECs cultured | BBB | 20 | Growth Factors | T10 | 1000000 cells | 7d | 42 | cephalotin sodium, carprofen, hartmans repl. fluid | Ketamin | Contusion |
| Guest, JD (12) | OECs cultured | BBB | 47 | Antibiotic | T9/T10 | 400000 cells | 5 min | 140 | cefaxolin sodium, tetraycline, vitamin C, baytril | Ketamin | Transection |
| Jia, B (13) | OECs cultured | BBB | 17 | unknown | T10 | 100000 cells | 7d | 56 | none | Ketamin | Transection |
| Keyvan-Fouladi, N (14) | OECs cultured | Directed forepaw reaching | 14 | Growth Factors | C2 | 25000000 cells | 56d | 56 | none | unknown | Hemisection |
| Lebedev, SV (15) | OECs cultured | BBB | 53 | Antibiotics+Growth Factors | T9 | 1500000 cells | 5 min | 56 | none | Ketamin | Contusion |
| Li, BC (16) | OECs cultured | BBB | 20 | Antibiotics+Growth Factors | T10 | 90000 cells | 7d | 42 | bicillin | Pentobarbital | Contusion |
| Li, BC (17) | OECs cultured | BBB | 10 | Antibiotics+Growth Factors | T10 | 90000 cells | 7d | 42 | bicillin | Pentobarbital | Contusion |
| Lopez-Vales, R (18) | OECs cultured | BBB | 21 16 | Antibiotics+Growth Factors | T8 | 1500000 cells 180000 cells | 30 min | 270 90 | none | Pentobarbital | Transection Photochemical |
| López-Vales, R (19) | OECs cultured | BBB | 16 | unknown | T8 | 1500000 cells | 45d | 195 | none | Pentobarbital | Transection |
| López-Vales, R (20) | OECs cultured | BBB | 13 10 | unknown | T8 | 1500000 cells | 30 min 7d | 270 | none | Pentobarbital | Transection |
| López-Vales, R (21) | OECs cultured | BBB | 10 | unknown | T8 | 180000 cells | 30 min | 14 | saline solution | Pentobarbital | Photochemical |
| Lu, J (22) | OLP OECs cultured | BBB | 14 15 | Antibiotics+Growth Factors | T10 | 5 pieces 100000 cells | 5 min | 56 | bupivacaine, carprofen, keflin, sodium chloride | Ketamin | Transection |
| Lu, J (23) | OLP | BBB | 12 | not cultured | T10 | 4 pieces | 28d | 98 | antibiotics | Ketamin | Transection |
| Masgutova, GA (24) | OECs cultured | BBB | 14 | unknown | T8 | 200000 cells | 5 min | 60 | none | Others | Hemisection |
| Moreno-Flores, M (25) | OECs cultured | Beam walking Tape sensing Tape removal | 8 8 8 | Growth Factors | C3 | 300000 cells | 5 min | 56 | none | Others | Compression |
| Nash, HH (26) | OECs cultured | Directed forepaw reaching | 14 | unknown | C3 | 200000 cells | 5 min | 35 | saline | Volatile | Transection |
| Pearse, DD (27) | OECs cultured | BBB | 24 | Growth Factors | T8 | 2000000 cells | 7d | 63 | gentamicin, buprenex | Ketamin | Contusion |
| Plant, GW (28) | OECs cultured | BBB | 13 16 | Antibiotics+Growth Factors | T9/T10 | 200000 cells | 30 min 7d | 56 | bicillin, ringer lactate, durafilm | Volatile | Contusion |
| Resnick, DK (29) | OECs cultured | BBB | 17 | Antibiotics+Growth Factors | T8/T9 | 250000 cells | 5 min | 42 | none | Others | Contusion |
| Salehi, M (30) | OECs cultured | BBB | 12 | Antibiotics+Growth Factors | T8/T9 | 1000000 cells | 9d | 37 | cefazolin, acetaminophen, cyclosporine, gentamicin | Ketamin | Compression |
| Sasaki, M (31) | OECs cultured | BBB | 26 | Growth Factors | T9 | 150000 cells | 5 min | 35 | none | Ketamin | Hemisection |
| Steward, O (32) | OLP | BBB | 9 7 | not cultured | T10 | 8 pieces | 28d | 118 99 | bupivacaine, carprofen buprenorphine, baytril | Ketamin | Transection |
| Sun, TS (33) | OECs | BBB | 30 30 | not cultured | T13 | 800000 cells | 5 min | 168 | none | Others | Transection |
| Takami, T (34) | OECs cultured | BBB | 42 | Antibiotics+Growth Factors | T9 | 2000000 cells | 7d | 77 | bicillin | Volatile | Contusion |
| Verdú, E (35) | OECs cultured | BBB | 16 | Growth Factors | T8 | 180000 cells | 30 min | 90 | amitriptyline | Pentobarbital | photochemical |
| Wang, G (36) | OECs cultured | BBB | 12 | Growth Factors | T9 | 1000000 cells | 7d | 84 | none | Chloral Hydrate | Hemisection |
| Wu,J (37) | OECs cultured | BBB | 16 | Antibiotics+Growth Factors | T9/T10 | 200000 cells | 5 min | 84 | physiological salt solution | Ketamin | Contusion |
| Wu, W (38) | OECs cultured | BBB | 16 | Antibiotics+Growth Factors | T11 | 100000 cells | 5 min | 28 | gentamicin, cyclosporin,ge-latin sponge | Chloral Hydrate | Hemisection |
| Xiao, M (39) | OECs cultured | spont. vertical expl. horizontal rope walking | 24 24 | Antibiotics+Growth Factors | C4 | 200000 cells | 7d | 77 | gelfoam, penicillin g, cyclosporineA | Ketamin | Hemisection |
| Yamamoto, M (40) | OM cultured | Directed forepaw reaching | 13 | Antibiotics+Growth Factors | C2 | 100000 cells | 56d | 126 | none | Others | Photochemical |
| Yan, HB (41) | OECs | BBB | 20 | not cultured | T10 | unknown | unknown | 56 | none | Others | Transection |
| Yazdani, SO (42) | OECs cultured | BBB | 14 | Growth Factors | T8/T9 | 1000000 cells | 7d | 42 | enrofloxacin | Unknown | Compression |
| Yu-Hai, M (43) | OECs cultured | BBB | 16 | Growth Factors | T9 | 100000 cells | 5 min | 56 | cefazolin sodium | Pentobarbital | Contusion |
| Yuan, PW (44) | OECs cultured | BBB | 20 | Antibiotics+Growth Factors | T10 | 100 cells | 5 min | 56 | none | Chloral Hydrate | Transection |
| Zhang, SX (45) | OLP OLP+OECs cultured | BBB | 16 15 | Growth Factors | T10 | 1 piece 6000000 cells | 42d | 154 | buprenorphine, cephazolin | Pentobarbital | Contusion |
| Zhang, J (46) | OECs cultured | BBB | 20 | Growth Factors | T10 | 600000 cells | 7d | 63 | bicillin, cyclosporin A | Pentobarbital | Contusion |
| Wang, L (47) | OECs cultured | BBB | 20 | unknown | T9-T11 | 1000000 cells | 5 min | 56 | unknown | Chloral Hydrate | Transection |
| Torres-Espín, A (48) | OECs cultured | BBB | 16 14 | Growth Factors | T8/9 | 450000 cells | 5 min 7d | 42 49 | amoxicillin | Ketamin | Contusion |
| Barbour, HR (49) | OECs cultured | BBB | 22 | Growth Factors | T10 | 500000 cells | 14d | 140 | penicillin, temgesic | Volatile | Contusion |

1. Amemori T, Jendelova P, Ruzickova K, Arboleda D, Sykova E. Co-transplantation of olfactory ensheathing glia and mesenchymal stromal cells does not have synergistic effects after spinal cord injury in the rat. Cytotherapy. 2010;12(2):212-25.

2. Aoki M, Kishima H, Yoshimura K, Ishihara M, Ueno M, Hata K, et al. Limited functional recovery in rats with complete spinal cord injury after transplantation of whole-layer olfactory mucosa: laboratory investigation. Journal of neurosurgery Spine. 2010;12(2):122-30.

3. Cao L, Liu L, Chen ZY, Wang LM, Ye JL, Qiu HY, et al. Olfactory ensheathing cells genetically modified to secrete GDNF to promote spinal cord repair. Brain : a journal of neurology. 2004;127(Pt 3):535-49.

4. Carrillo-Ruiz JD, Andrade P, Silva F, Vargas G, Maciel-Navarro MM, Jimenez-Botello LC. Olfactory bulb implantation and methylprednisolone administration in the treatment of spinal cord injury in rats. Neuroscience letters. 2009;462(1):39-44.

5. Centenaro LA, Jaeger MD, Ilha J, de Souza MA, Kalil-Gaspar PI, Cunha NB, et al. Olfactory and respiratory lamina propria transplantation after spinal cord transection in rats: Effects on functional recovery and axonal regeneration. Brain Res. 2011;1426:54-72.

6. Chen H, Zheng X, Sheng W, Wei Q, Jiang T, Jin G. Transplantation of low-power laser-irradiated olfactory ensheathing cells to promote repair of spinal cord injury in rats. Neural Regeneration Research. 2009;4(3):171-7.

7. Collazos-Castro JE, Muneton-Gomez VC, Nieto-Sampedro M. Olfactory glia transplantation into cervical spinal cord contusion injuries. Journal of neurosurgery Spine. 2005;3(4):308-17.

8. Deng YB, Liu Y, Zhu WB, Bi XB, Wang YZ, Ye MH, et al. The co-transplantation of human bone marrow stromal cells and embryo olfactory ensheathing cells as a new approach to treat spinal cord injury in a rat model. Cytotherapy. 2008;10(6):551-64.

9. Garcia-Alias G, Lopez-Vales R, Fores J, Navarro X, Verdu E. Acute transplantation of olfactory ensheathing cells or Schwann cells promotes recovery after spinal cord injury in the rat. Journal of neuroscience research. 2004;75(5):632-41.

10. García-Alías G, López-Vales R, Verdú E, Navarro X, Suso S, Forés J. El trasplante de células de la glía envolvente del bulbo olfatorio tras lesión de la médula espinal: Estudio experimental en ratas. TITLEREVISTA. 2005;49(04):301-6.

11. Gorrie CA, Hayward I, Cameron N, Kailainathan G, Nandapalan N, Sutharsan R, et al. Effects of human OEC-derived cell transplants in rodent spinal cord contusion injury. Brain Res. 2010;1337:8-20.

12. Guest JD, Herrera L, Margitich I, Oliveria M, Marcillo A, Casas CE. Xenografts of expanded primate olfactory ensheathing glia support transient behavioral recovery that is independent of serotonergic or corticospinal axonal regeneration in nude rats following spinal cord transection. Experimental neurology. 2008;212(2):261-74.

13. Jia B, Li J, He XJ, Yu JL, Zhao ZJ. Combination of olfactory ensheathing cells from the olfactory mucosa and muscle basal lamina for the treatment of spinal cord injury. [Chinese]. Journal of Clinical Rehabilitative Tissue Engineering Research. 2008;12(51):10041-4.

14. Keyvan-Fouladi N, Raisman G, Li Y. Functional repair of the corticospinal tract by delayed transplantation of olfactory ensheathing cells in adult rats. The Journal of neuroscience : the official journal of the Society for Neuroscience. 2003;23(28):9428-34.

15. Lebedev SV, Karasev AV, Chekhonin VP, Savchenko EA, Viktorov IV, Chelyshev YA, et al. Study of the efficiency of transplantation of human neural stem cells to rats with spinal trauma: the use of functional load tests and BBB test. Bulletin of experimental biology and medicine. 2010;149(3):377-82.

16. Li BC, Li Y, Chen LF, Chang JY, Duan ZX. Olfactory ensheathing cells can reduce the tissue loss but not the cavity formation in contused spinal cord of rats. Journal of the neurological sciences. 2011;303(1-2):67-74.

17. Li BC, Xu C, Zhang JY, Li Y, Duan ZX. Differing Schwann cells and olfactory ensheathing cells behaviors, from interacting with astrocyte, produce similar improvements in contused rat spinal cord's motor function. Journal of molecular neuroscience : MN. 2012;48(1):35-44.

18. Lopez-Vales R, Fores J, Navarro X, Verdu E. Olfactory ensheathing glia graft in combination with FK506 administration promote repair after spinal cord injury. Neurobiology of disease. 2006;24(3):443-54.

19. Lopez-Vales R, Fores J, Navarro X, Verdu E. Chronic transplantation of olfactory ensheathing cells promotes partial recovery after complete spinal cord transection in the rat. Glia. 2007;55(3):303-11.

20. Lopez-Vales R, Fores J, Verdu E, Navarro X. Acute and delayed transplantation of olfactory ensheathing cells promote partial recovery after complete transection of the spinal cord. Neurobiology of disease. 2006;21(1):57-68.

21. Lopez-Vales R, Garcia-Alias G, Guzman-Lenis MS, Fores J, Casas C, Navarro X, et al. Effects of COX-2 and iNOS inhibitors alone or in combination with olfactory ensheathing cell grafts after spinal cord injury. Spine. 2006;31(10):1100-6.

22. Lu J, Feron F, Ho SH, Mackay-Sim A, Waite PME. Transplantation of nasal olfactory tissue promotes partial recovery in paraplegic adult rats. Brain Res. 2001;889(1-2):344-57.

23. Lu J, Feron F, Mackay-Sim A, Waite PM. Olfactory ensheathing cells promote locomotor recovery after delayed transplantation into transected spinal cord. Brain : a journal of neurology. 2002;125(Pt 1):14-21.

24. Masgutova GA, Savchenko EA, Viktorov IV, Masgutov RF, Chelyshev YA. Reaction of oligoglia to spinal cord injury in rats and transplantation of human olfactory ensheathing cells. Bulletin of experimental biology and medicine. 2010;149(1):135-9.

25. Moreno-Flores MT, Bradbury EJ, Martin-Bermejo MJ, Agudo M, Lim F, Pastrana E, et al. A clonal cell line from immortalized olfactory ensheathing glia promotes functional recovery in the injured spinal cord. Molecular therapy : the journal of the American Society of Gene Therapy. 2006;13(3):598-608.

26. Nash HH, Borke RC, Anders JJ. Ensheathing cells and methylprednisolone promote axonal regeneration and functional recovery in the lesioned adult rat spinal cord. The Journal of neuroscience : the official journal of the Society for Neuroscience. 2002;22(16):7111-20.

27. Pearse DD, Sanchez AR, Pereira FC, Andrade CM, Puzis R, Pressman Y, et al. Transplantation of Schwann cells and/or olfactory ensheathing glia into the contused spinal cord: Survival, migration, axon association, and functional recovery. Glia. 2007;55(9):976-1000.

28. Plant GW, Christensen CL, Oudega M, Bunge MB. Delayed transplantation of olfactory ensheathing glia promotes sparing/regeneration of supraspinal axons in the contused adult rat spinal cord. Journal of neurotrauma. 2003;20(1):1-16.

29. Resnick DK, Cechvala CF, Yan Y, Witwer BP, Sun D, Zhang S. Adult olfactory ensheathing cell transplantation for acute spinal cord injury. Journal of neurotrauma. 2003;20(3):279-85.

30. Salehi M, Pasbakhsh P, Soleimani M, Abbasi M, Hasanzadeh G, Modaresi MH, et al. Repair of spinal cord injury by co-transplantation of embryonic stem cell-derived motor neuron and olfactory ensheathing cell. Iranian biomedical journal. 2009;13(3):125-35.

31. Sasaki M, Lankford KL, Zemedkun M, Kocsis JD. Identified olfactory ensheathing cells transplanted into the transected dorsal funiculus bridge the lesion and form myelin. The Journal of neuroscience : the official journal of the Society for Neuroscience. 2004;24(39):8485-93.

32. Steward O, Sharp K, Selvan G, Hadden A, Hofstadter M, Au E, et al. A re-assessment of the consequences of delayed transplantation of olfactory lamina propria following complete spinal cord transection in rats. Experimental neurology. 2006;198(2):483-99.

33. Sun TS, Ren JX, Shi JG. [Repair of acute spinal cord injury promoted by transplantation of olfactory ensheathing glia]. Zhongguo yi xue ke xue yuan xue bao Acta Academiae Medicinae Sinicae. 2005;27(2):143-7.

34. Takami T, Oudega M, Bates ML, Wood PM, Kleitman N, Bunge MB. Schwann cell but not olfactory ensheathing glia transplants improve hindlimb locomotor performance in the moderately contused adult rat thoracic spinal cord. The Journal of neuroscience : the official journal of the Society for Neuroscience. 2002;22(15):6670-81.

35. Verdu E, Garcia-Alias G, Fores J, Lopez-Vales R, Navarro X. Olfactory ensheathing cells transplanted in lesioned spinal cord prevent loss of spinal cord parenchyma and promote functional recovery. Glia. 2003;42(3):275-86.

36. Wang G, Ao Q, Gong K, Zuo H, Gong Y, Zhang X. Synergistic effect of neural stem cells and olfactory ensheathing cells on repair of adult rat spinal cord injury. Cell transplantation. 2010;19(10):1325-37.

37. Wu J, Sun TS, Ren JX, Wang XZ. Ex vivo non-viral vector-mediated neurotrophin-3 gene transfer to olfactory ensheathing glia: effects on axonal regeneration and functional recovery after implantation in rats with spinal cord injury. Neuroscience bulletin. 2008;24(2):57-65.

38. Wu WJ, Hui GZ, Lu RB, Miao ZN. Combined transplantation of human fetal olfactory ensheathing cells and rat embryonic spinal cord tissues in the treatment of spinal cord injury in rats. Chinese Journal of Clinical Rehabilitation. 2006;10(41):175-9.

39. Xiao M, Klueber KM, Lu C, Guo Z, Marshall CT, Wang H, et al. Human adult olfactory neural progenitors rescue axotomized rodent rubrospinal neurons and promote functional recovery. Experimental neurology. 2005;194(1):12-30.

40. Yamamoto M, Raisman G, Li D, Li Y. Transplanted olfactory mucosal cells restore paw reaching function without regeneration of severed corticospinal tract fibres across the lesion. Brain Res. 2009;1303:26-31.

41. Yan HB, Zhang ZM, Jin DD, Wang XJ, Lu KW. [The repair of acute spinal cord injury in rats by olfactory ensheathing cells graft modified by glia cell line-derived neurotrophic factor gene in combination with the injection of monoclonal antibody IN-1]. Zhonghua wai ke za zhi [Chinese journal of surgery]. 2009;47(23):1817-20.

42. Yazdani SO, Pedram M, Hafizi M, Kabiri M, Soleimani M, Dehghan MM, et al. A comparison between neurally induced bone marrow derived mesenchymal stem cells and olfactory ensheathing glial cells to repair spinal cord injuries in rat. Tissue & cell. 2012;44(4):205-13.

43. Yu-Hai M, Zhang Y, Cao L, Su JC, Wang ZW, Xu AB, et al. Effect of neurotrophin-3 genetically modified olfactory ensheathing cells transplantation on spinal cord injury. Cell transplantation. 2010;19(2):167-77.

44. Yuan PW, He XJ, Wang GY, Hao YQ, Liu DY. Olfactory ensheathing cell transplantation and inhibition of NogoA, NgR and RhoA expression in the damaged zone to ameliorate spinal cord injury. Neural Regeneration Research. 2010;5(13):999-1003.

45. Zhang SX, Huang F, Gates M, Holmberg EG. Scar ablation combined with LP/OEC transplantation promotes anatomical recovery and P0-positive myelination in chronically contused spinal cord of rats. Brain Res. 2011;1399:1-14.

46. Zhang J, Liu Z, Chen H, Duan Z, Zhang L, Chen L, et al. Synergic Effects of EPI-NCSCs and OECs on the Donor Cells Migration, the Expression of Neurotrophic Factors, and Locomotor Recovery of Contused Spinal Cord of Rats. Journal of molecular neuroscience : MN. 2014.

47. Wang L, Yang P, Liang X, Ma L, Wei J. [Comparison of therapeutic effects of olfactory ensheathing cells derived from olfactory mucosa or olfactory bulb on spinal cord injury mouse models]. Xi bao yu fen zi mian yi xue za zhi = Chinese journal of cellular and molecular immunology. 2014;30(4):379-83.

48. Torres-Espin A, Redondo-Castro E, Hernandez J, Navarro X. Bone marrow mesenchymal stromal cells and olfactory ensheathing cells transplantation after spinal cord injury--a morphological and functional comparison in rats. The European journal of neuroscience. 2014;39(10):1704-17.

49. Barbour HR, Plant CD, Harvey AR, Plant GW. Tissue sparing, behavioral recovery, supraspinal axonal sparing/regeneration following sub-acute glial transplantation in a model of spinal cord contusion. BMC neuroscience. 2013;14:106.
