# Supplementary material for: Olfactory Ensheathing Cell Transplantation in Experimental Spinal Cord Injury: Effect size and Reporting Bias of 62 Experimental Treatments: A Systematic Review and Meta-Analysis
Source: PLoS Biol. 2016 May 31;14(5):e1002468. doi: 10.1371/journal.pbio.1002468 (PMC4886956; doi:10.1371/journal.pbio.1002468)
Supplement: S1 Text — (DOCX) [file pbio.1002468.s004.docx]

**S2 Text**

1. ${ES}_{i}=100\% \times\frac{\left( \bar{x}_{c}-\bar{x}_{sham} \right)-(\bar{x}_{rx}-\bar{x}_{sham})}{\bar{x}_{c}-\bar{x}_{sham}}$

(2) ${SE}_{i}=\sqrt{\frac{{{SD}^{2}}_{c*}}{n_{c}}+\frac{{{SD}^{2}}_{rx*}}{n_{rx}}}$

(3) ${SD}_{c*}=100\times\frac{{SD}_{c}}{\bar{x}_{c}-\bar{x}_{sham}}$ and ${SD}_{rx*}=100\times\frac{{SD}_{rx}}{\bar{x}_{rx}-\bar{x}_{sham}}$

1. Normalised mean difference / effect size: where $\bar{x}_{c}$ , $\bar{x}_{rx}$ and $\bar{x}_{sham}$ represent the mean outcomes in the control, treatment and sham group
2. Corresponding standard error: where $n_{c}$ and $n_{rx}$ represent the number of animals in the control and treatment group; SD^2^_c*_ and SD^2^_rx*_ refer to the normalized standard deviations from the control and treatment group (see equation 3)
3. Normalized standard deviations: where $\bar{x}_{c}$ , $\bar{x}_{rx}$ and $\bar{x}_{sham}$ represent the mean outcomes in the control, treatment and sham group
